# Visual Adaptation to Thin and Fat Bodies Transfers across Identity

**DOI:** 10.1371/journal.pone.0043195

**Published:** 2012-08-15

**Authors:** Dennis Hummel, Anne K. Rudolf, Karl-Heinz Untch, Ralph Grabhorn, Harald M. Mohr

**Affiliations:** 1 Department of Neurocognitive Psychology, Institute of Psychology, Goethe University, Frankfurt, Germany; 2 Department of Psychiatry, Psychosomatics and Psychotherapy, Goethe University Hospital, Frankfurt, Germany; Royal Holloway, University of London, United Kingdom

## Abstract

Visual perception is highly variable and can be influenced by the surrounding world. Previous research has revealed that body perception can be biased due to adaptation to thin or fat body shapes. The aim of the present study was to show that adaptation to certain body shapes and the resulting perceptual biases transfer across different identities of adaptation and test stimuli. We designed two similar adaptation experiments in which healthy female participants adapted to pictures of either thin or fat bodies and subsequently compared more or less distorted pictures of their own body to their actual body shape. In the first experiment (n = 16) the same identity was used as adaptation and test stimuli (i.e. pictures of the participant’s own body) while in the second experiment (n = 16) we used pictures of unfamiliar thin or fat bodies as adaptation stimuli. We found comparable adaptation effects in both experiments: After adaptation to a thin body, participants rated a thinner than actual body picture to be the most realistic and vice versa. We therefore assume that adaptation to certain body shapes transfers across different identities. These results raise the questions of whether some type of natural adaptation occurs in everyday life. Natural and predominant exposure to certain bodily features like body shape – especially the thin ideal in Western societies – could bias perception for these features. In this regard, further research might shed light on aspects of body dissatisfaction and the development of body image disturbances in terms of eating disorders.

## Introduction

Visual perception is strongly influenced by experience and the stimuli that surround us. More specifically, visual perception can be altered by adaptation to a certain stimulus or a certain class of stimuli. This prolonged exposure – especially in terms of psychophysical experiments – usually leads to a perceptual aftereffect, biasing perception into the opposite direction of the adaptation stimulus. Aftereffects for low-level visual properties have been known for a relatively long time [1], [2], [3].

Moreover, there is a growing body of literature about so-called higher order aftereffects and face aftereffects have been the target of many studies. It has been shown that face aftereffects can be observed for certain specific facial properties like gender [4], ethnicity [4], [5], emotional expression [4], [6], or even gaze direction [7], illustrating the specificity of higher-order aftereffects. For example, after adaptation to female faces, subsequently seen gender-neutral faces appear male to the observer and vice versa. These higher order aftereffects transfer across changes in stimulus size [8] or stimulus orientation [9] and thus are vastly invariant to these low-level stimulus properties. This invariance supports the assumption that face aftereffects cannot be solely explained by low-level visual properties but are essentially evoked by complex visual stimuli like faces and their corresponding features like facial expression. This indicates that it is not only neurons coding for low-level visual properties that change their response pattern due to adaptation but also and essentially neurons coding for complex visual stimuli and their specific features.

Strikingly, face adaptation also – at least partly – transfers across different identities, as has been shown for figural aftereffects [10], age [11] and extensively for facial expression of emotions [6], [12], [13], [14]. Here, using faces from different individuals as adaptation and test stimuli evoked measurable aftereffects, although these effects are significantly weaker than when using the same individual face as adaptation and test stimuli. The results of these studies suggest identity-dependent as well as identity-independent representations of faces [15], [7]. Fox et al. [16] revealed an interesting asymmetry between identity and facial expression by showing that identity aftereffects completely transfer across different facial expressions. However, using an interesting methodological approach, Benton [17] could show that facial expression aftereffects are profoundly decreased when photographic negation is applied to the adaptation stimuli, thus supporting the idea that the visual representation of facial expression incorporates surface information rather than edge information.

Face adaptation successfully demonstrates that aftereffects are not restricted to low-level properties of visual stimuli. While faces convey much information about an observed person, the same holds true for the rest of the body. Given this importance of body perception and its contribution to social communication and interaction, there have been studies investigating adaptation to predominantly bodily properties, i.e. thin and fat body shapes. In a former study of our workgroup, participants adapted to distorted thin and fat pictures of their own bodies and subsequently rated pictures of their own bodies [18]. We found that after adapting to a thin picture of their own body participants judged a thinner than actual body picture to be the most realistic and vice versa, demonstrating a perceptual bias due to adaptation to certain body shapes.

Winkler and Rhodes [19] had their participants adapt to representations of either thin or fat unfamiliar bodies and asked them to rate distorted unfamiliar bodies in terms of attractiveness and normality before and after adaptation. They found that the most attractive and most normal appearing bodies became thinner after adaptation to thin bodies. The most normal looking body changed after adaptation to fat bodies, while the most attractive body did not. Glauert et al. [20] could replicate these findings using more realistic body pictures. Furthermore, they found that greater body dissatisfaction and internalization of the thin western ideal were related to a thinner most normal and ideal body, a greater discrepancy between most normal and most ideal body, and a reduced effect for adaptation to fat bodies. These results show, that 1) body perception can be easily manipulated via adaptation and that 2) body perception and the magnitude of adaptation to bodies are related to body dissatisfaction. Importantly, the aforementioned studies were restricted to pictures of unfamiliar bodies as adaptation and test stimuli – i.e. distorted photographs in Winkler and Rhodes [19] and 3D models representing certain body mass indexes in Glauert et al. [20] – and thus did not test for an identity invariance of body shape after­effects. As identity invariance in adaptation to other specific stimulus properties has been shown for faces (see above), the question remains whether identity invariance can also be found in adaptation to specific bodily properties like body shape.

In a review by Minnebusch and Daum [21] the authors outlined some important similarities for face and body perception. Both stimulus categories demand processes which involve the perception of relations among features of a stimulus (configural processing) [22], while it is yet unknown whether this processing in face and body perception shares the same mechanism. Furthermore, faces and bodies activate specific cortical regions. While faces activate the occipital face area (OFA) and the fusiform face area (FFA, [23]), activation for bodies is found in the extrastriate body area (EBA, [24]) and the fusiform body area (FBA, [25]). Both stimulus categories additionally activate the superior temporal sulcus (STS). Haxby et al. [15] have suggested that face perception activates a core system (OFA, FFA and STS) which processes invariant face aspects (e.g. identity and ethnicity) and an extended system which processes variable facial features (e.g. emotions, gaze direction). A similar functional system for the analysis of visual body appearance might be represented by EBA, FBA and STS [26], whereas the analysis of information about emotions and intentions recruits additional brain regions [27], [28]. Furthermore, neural adaptation to certain body shapes can be found in the FBA [18].

The perception of the own body shape is of high significance in terms of social comparison and body (dis-)satisfaction. A biased body shape perception also plays a central role in certain psychiatric diseases like eating disorders. Patients suffering from Anorexia nervosa or Bulimia nervosa overestimate their own body size [29], which is why this distorted body image also is one of the main diagnostic criteria in the DSM-IV TR [30]. Moreover, eating disordered patients show a severe overconcern with body size and shape and an elevated attention towards disorder relevant stimuli like bodies [31]. So-called “thinspiration" websites are a force to be reckoned with and emphasize patients’ urge and motivation to occupy themselves with specific body shapes, i.e. pictures of thin models which are furthermore often manipulated to appear even thinner. In light of these findings and the fact that perception of the own body can be easily manipulated by adaptation to certain stimuli [18], it might be speculated whether a prolonged an intentional but ‘everyday-life’ exposure to certain body shapes can alter the way a person perceives and rates her or his own body shape.

The aim of the present study was to show that adaptation to certain body shapes transfers across identities and that the perception and judgment of the own body can be altered due to prolonged inspection of unfamiliar bodies. To this end, we designed two similar experiments, using the same paradigm and task but different adaptation stimuli. A similar behavioral experiment has already been published in Hummel et al. [18]. In the first experiment we used pictures from the same individuals as adaptation and test stimuli, i.e. distorted digital pictures of the participants’ own bodies. In the second experiment participants adapted to pictures of demonstratively thin or corpulent other women and subsequently conducted a binary judgment (thinner or fatter than their real body) on more or less distorted pictures of their own bodies as test stimuli. We hypothesized to find significant aftereffects in both experiments, demonstrating an adaptation transfer across identities for body shape aftereffects. As dissatisfaction with the own body shape is of high prevalence for women in Western societies [32], we decided to restrict our samples to female participants.

## Experiment 1

### Materials and Methods

#### Ethics statement

All experiments were approved by the ethics commission of the Goethe-University medical school and are in compliance with the Declaration of Helsinki. Informed consent was obtained from all participants of experiments 1 and 2. Written informed consent was obtained from all participants.

#### Participants

Sixteen healthy adult female volunteers were recruited and participated in experiment 1. The mean age was 21.7 years (SD = 2.0, range = 20–27 years). For each participant the Body Mass Index (BMI) was calculated which resulted in a mean BMI of 19.8 (SD = 1.8). All participants had normal or corrected-to-normal vision.

#### Stimuli

A digital photograph of each participant was created on a monotonous, white-colored background in a standardized pose (standing upright, feet approximately as wide as the shoulders, arms spread horizontally). The photograph showed the participant’s whole body but omitted the head. Feet and forearms were also cropped as these body parts were not manipulated by the software (see below). Participants wore standardized clothes, namely a black t-shirt and black leggings. The clothes were available in several sizes to make sure they would fit tightly to the body shape.

The computer program “Body Form Imaging" [33] was used to manipulate the digital picture of each participant. Shoulders, chest, belly, hips, thighs and calves were jointly distorted in several steps to obtain 21 pictures with different degrees of body size relative to the original. For each subject, we created the following pictures: ten fatter than actual pictures (distortion at +1 to +10 steps of the original picture) and ten thinner than actual pictures (distortion at -1 to -10 steps of the original picture) as well as the original picture.

**Figure 1:**
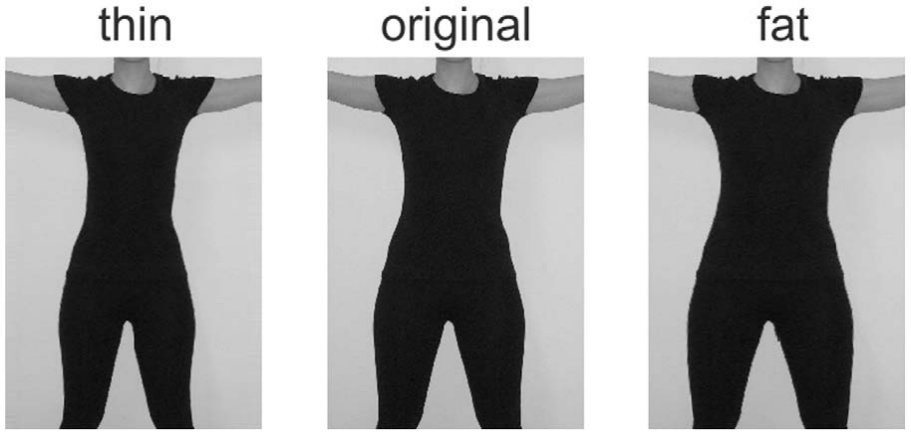
Examples for the adaptation stimuli in comparison to the original picture.

For these body pictures one step resembles ∼1% increase or decrease of the body surface shown in a picture. To maintain a realistic appearance of the body shape (pictures were not simply stretched or compressed and both legs had to be manipulated individually), the used software package for manipulating the body pictures could not provide pictures with a distinct step width for the body shape as a whole (i.e. an exact percentage of surface increase or decrease). Nonetheless, the stimuli used in the present study have been proven to be useful and adequate stimuli in previous experiments [18], [34], [35]. Figure 1 displays the adaptation stimuli as well as the original picture of one sample participant.

#### Experimental design

Each participant completed two adaptation sessions. The adaptation stimuli were the participant's own body pictures at +8 steps distortion (fatter than actual) or -8 steps distortion (thinner than actual), respectively. Between these two sessions there always was a break of at least one week to minimize possible adaptation effects from the first session during the second session. The order of adaptation sessions was counter-balanced.

Each session started with an adaptation of 240 sec in which the adaptation stimulus was constantly presented. In the following, we will refer to this adaptation as the initial adaptation. The participants were instructed to watch their own body pictures attentively, without any restrictions to eye movements.

**Figure 2:**
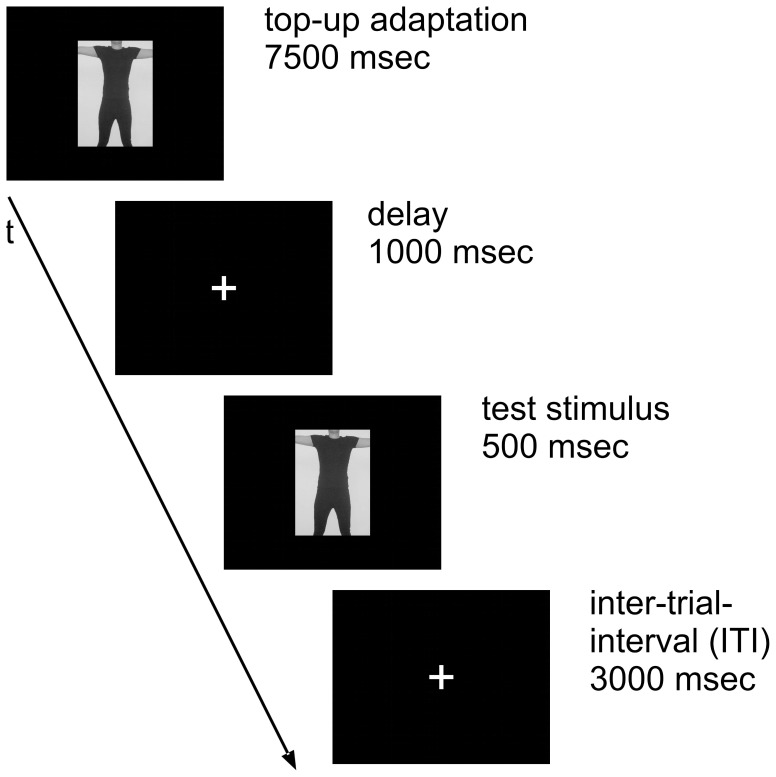
Experimental design of a trial in experiment 1. Shown are the elements of a single trial and their corresponding durations (here exemplary for adaptation to a thin body picture).

After the initial adaptation the task started, consisting of 50 trials which were separated by an inter-trial-interval (ITI) of 3000 msec. The sequence of a trial of experiment 1 is also shown in figure 2.

Each trial started with a top-up adaptation of 7500 msec using the same adaptation stimulus as in the initial adaptation. After the top-up adaptation and an inter-stimulus-interval (ISI) of 1000 msec, another picture from the pool of 21 distorted body pictures was shown for 500 msec as a test stimulus. Using a staircase paradigm, these 21 body pictures were integrated into two alternating staircases, one starting with the body picture at +8 steps distortion and the other starting with the body picture at -8 steps distortion. Each staircase had a constant step size of one step of distortion. After each test stimulus the participants judged whether they thought their body to be fatter or thinner than the test stimulus by pressing one of two response buttons. The picture shown next by a staircase depended on the previous response, given by the participant (i.e. if a participant was presented with picture -6 steps and responded to be actually fatter, then next time this staircase would show picture -5 steps and so on). If a participant constantly responds with the same answer for several trials and then her response changes at a certain picture, a so-called turning point is defined. By taking the turning points and their corresponding pictures from the raw data, we could identify the pictures which were judged to be the most realistic by the participants. The whole procedure (240 sec initial adaptation +50 trials) was completed two times during each of both adaptation sessions (thin and fat).

Due to our hypothesis, after adaptation to a thinner than actual body picture participants should experience a perceptual bias in the opposite direction of the adaptation stimulus and thus perceive all subsequently shown test stimuli as fatter than they really are and consequently judge a thinner than actual test stimulus to be the most realistic and vice versa. Thus, the pictures judged to be the most realistic ones should differ significantly as a result of adaptation direction and turning points should generally correspond to thinner body pictures after adaptation to the thin body picture than after adaptation to the fat body picture.

Both experiments were programmed and presented using “Presentation" (Neurobehavioral Systems, Albany, USA). A 17″ CRT computer monitor was used for visual output (resolution: 1024×768 pixels). Stimuli were presented with a viewing angle of about 9.7° in height and a viewing angle of about 6.2° in width on the screen with black background. Participants viewed the computer screen from a distance of about 65 cm. A regular computer keyboard with two designated response buttons was used as input device.

### Results and Discussion

**Figure 3:**
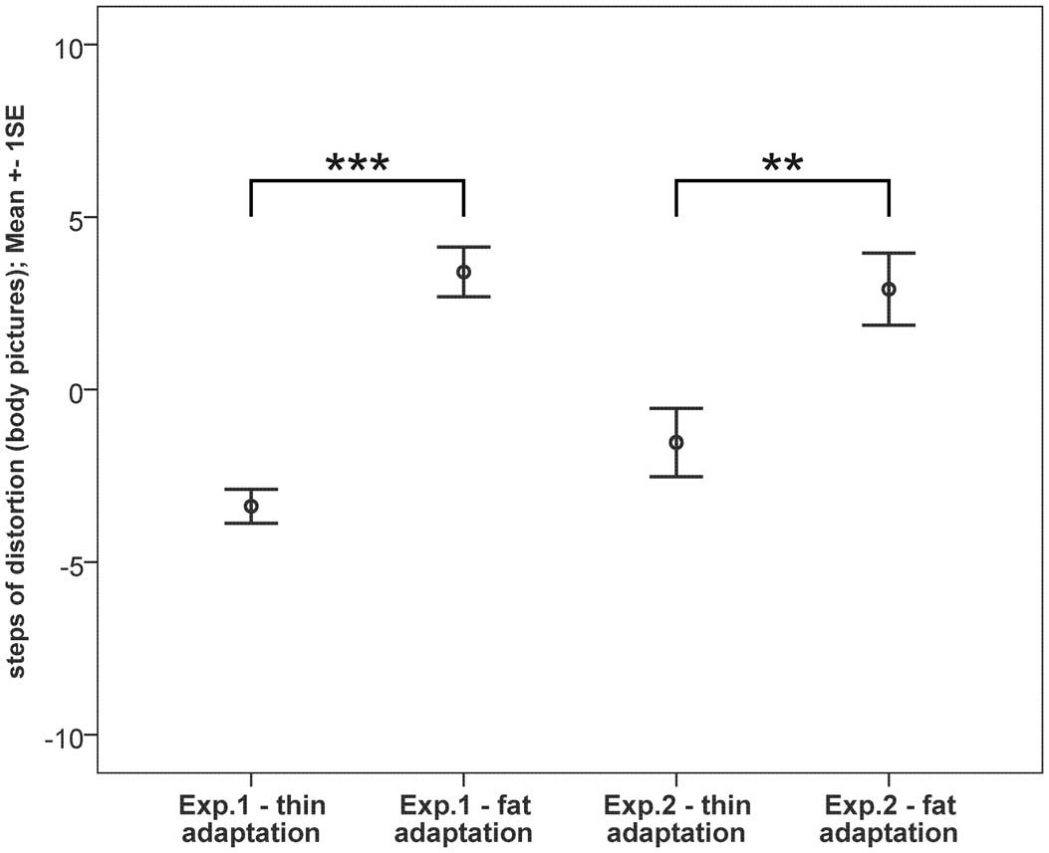
Results of both experiments. Shown are the ratings of the participants of experiment 1 (same identity, left side) and experiment 2 (different identities, right side) for adaptation directions. Adaptation to a thin body shape resulted in a thinner than actual body picture to be rated as the most realistic and vice versa.

We obtained the median for the turning points of each adaptation session from each participant, resulting in two median values, one for each adaptation condition, for each of the 16 participants. As Kolmogorov-Smirnov tests turned out to be not significant (both p>0.62), we assumed normal distribution and used parametric statistics for further analyses. The mean of the 16 medians for the thin adaptation condition was −3.4 steps (SD = 2.0), indicating that the participants judged a picture on which they were actually depicted thinner, to be the most realistic. For the fat adaptation condition the mean of the 16 medians was +3.4 steps (SD = 2.9), indicating that the participants judged a picture on which they were actually depicted fatter, to be the most realistic. A t-test for paired samples revealed a significant difference for thin adaptation vs. fat adaptation (n = 16, t = −11.463, p<.001). There were no correlations between BMI and the results of the adaptation conditions. The results are shown on the left side of figure 3.

In experiment 1 (same identity for adaptation and test stimuli), we clearly demonstrated that a psychophysical adaptation changes a participant’s judgment of her or his own body pictures with regard to the body shape. As expected, the design used in experiment 1 sufficiently induces a body shape aftereffect, as has earlier been shown in a similar experiment [18]. After adapting to a picture of their own thin body, participants perceive subsequently shown pictures of their own bodies as being fatter than actual and vice versa. Thus, due to this perceptual bias in the opposite direction of the adaptation stimulus (negative aftereffect), a thinner or fatter than actual picture of the own body is perceived as the most realistic picture, dependent on adaptation direction. While Winkler and Rhodes [19] and Glauert et al. [20] used pictures of other people’s bodies as adaptation and test stimuli, in the present study we complement these findings by showing that similar effects occur if pictures of the participant’s own bodies are used as adaptation and test stimuli.

## Experiment 2

### Materials and Methods

#### Participants

Sixteen healthy adult female volunteers were recruited and participated in experiment 2. One of these participants had formerly also completed experiment 1. The mean age was 23.1 years (SD = 3.5, range = 19–31 years). The mean BMI was 21.0 (SD = 1.3). The samples of both experiments did not significantly differ in age (N = 32, t = −1.368, p = .181) but in BMI (N = 32, t = −2.256, p = .031), indicating a lower BMI for the sample of experiment 1. All participants had normal or corrected-to-normal vision.

#### Stimuli

Again, we used body photographs as test stimuli for the judgment of the participants similar to those used in experiments 1 (ten fatter pictures, ten thinner pictures, and the original picture). The average step width again was ∼1%. Furthermore, in experiment 2, we used digital pictures showing the bodies (without heads) of 12 demonstratively corpulent (fat adaptation) and 12 demonstratively slim (thin adaptation) women, as adaptation stimuli. These pictures were taken mainly from advertisement. The women depicted wore underwear or bathing suits. The background of each picture was set white, similar to the pictures taken from the participants. Each body was presented in grayscale to prevent elevated attention towards colorful clothing of the depicted women.

#### Experimental design

Basically, we used the same experimental design as in experiment 1, except for substituting the adaptation stimuli. This time, during the initial adaptation participants were presented with body pictures of 12 slim or corpulent unfamiliar women, respectively. Each initial adaptation lasted for 216 sec showing each of the 12 adaptation stimuli three times for 6000 msec each in a pseudo-randomised sequence. In the top-up adaptation, an adaptation stimulus was randomly taken from the pool of 12 adaptation stimuli and was presented for 6000 msec. After the top-up adaptation and an ISI of 1000 msec, participants had to judge their own body pictures as test stimuli, which again were presented for 500 msec. Trials were separated by an ITI of 3000 msec. The order of adaptation sessions was counter-balanced. In experiment 2 we tested for the same hypothesis as in experiment 1.

Additionally, all participants completed a rating for the adaptation stimuli in which they had to rate each woman's attractiveness on a scale from 1 to 6 (1 = very unattractive, 6 = very attractive) and body weight on a scale from 1 to 6 (1 = very underweight, 6 = very overweight). This additional task was accomplished to control for dissimilarity of both adaptation categories (i.e. that thin women were perceived as significantly thinner than fat women) and to obtain information about a possible influence of perceived attractiveness of the adaptation stimuli. This rating was completed prior to one of the adaptation experiments (counter-balanced).

### Results and Discussion

We obtained the median for the turning points of each adaptation session from each participant, resulting in two median values, one for each adaptation condition, for each of the 16 participants. As Kolmogorov-Smirnov tests turned out to be not significant (both p>0.73), we again assumed normal distribution and used parametric statistics for further analyses. The mean for the 16 medians of the thin adaptation condition was −1.5 steps (SD = 3.9), indicating that the participants judged a picture on which they were actually depicted thinner to be the most realistic. For the fat adaptation condition the overall median was +2.9 steps (SD = 4.2), indicating that the participants judged a picture on which they were actually depicted fatter to be the most realistic. A t-test for paired samples revealed a significant difference for thin adaptation vs. fat adaptation (n = 16, t = −4.176, p = .001). There were no correlations between BMI and the results of the adaptation conditions. The results are shown on the right side of figure 3.

For the attractiveness rating of the adaptation stimuli the overall median was at 4.25 (range = 1.5 to 6) for slim women and at 2.5 (range = 1 to 4) for corpulent women. A Wilcoxon test revealed a significant difference between these two classes of adaptation stimuli (n = 16, Z = −3.417, p = .001), indicating that slim women were rated as being more attractive than corpulent women. For the body weight rating of the adaptation stimuli the median was at 2 (range = 1 to 3) for slim women and at 5.5 (range = 4.5 to 6) for corpulent women. A Wilcoxon test revealed a significant difference between these two classes of adaptation stimuli (n = 16, Z = −3.537, p<.001), indicating that corpulent women were rated as being heavier than slim women, thus confirming a significant dissimilarity of our adaptation stimuli for the stimulus property in question, i.e. perceived body shape. There were no significant correlations between a participant’s attractiveness or body weight rating of the adaptation stimuli and the rating of her own body pictures (all p>0.1).

In experiment 2 (different identities for adaptation and test stimuli), the direction of adaptation altered the participants’ decisions comparable to the results of experiment 1. This clearly shows that adaptation to certain body shapes and the resulting perceptual bias transfer across different identities. Remarkably, the adaptation transfer is emphasized by the fact that we used pictures of several unfamiliar bodies as adaptation stimuli, ruling out an effect solely based on one certain adaptation stimulus, i.e. one certain individual. As the body weight rating of the adaptation stimuli was significantly different for both adaptation categories, this supports the presumption that our adaptation stimuli were adequate to evoke oppositional aftereffects.

## Discussion

The results are in accordance with studies in the field of face adaptation, where adaptation effects were found to transfer across identities [6], [10], [11], [12], [13], [14], although these effects were generally less strong compared to conditions where adaptation and test stimuli had the same identity. For future research it would be interesting to test whether properties like body shape and identity are processed independently or whether adaptation to body shapes also interacts with certain other properties of a body (like identity and/or gender). Similar results have been shown for face adaptation [36], indicating that multiple dimensions (i.e. gender and ethnicity) are represented independently.

It is quite noticeable that the intentional inspection of other women’s bodies changes the way a woman judges pictures of her own body. As perceived attractiveness of the adaptation stimuli significantly differed for both categories, it might be possible that perceived attractiveness contributes to the adaptation effect. However, the magnitudes of the adaptation effects found in experiment 2 were not correlated to the magnitudes of the attractiveness ratings. In future studies it would be interesting to see whether the same adaptation effects occur when perceived attractiveness is the same for both adaptation categories.

Adaptation renormalizes perception, so that a ‘neutral point’ (i.e. what looks normal or real for each person individually) is shifted towards the adaptation stimulus. This opens the discussion about a possible adaptation to certain body shapes in everyday life. In terms of face adaptation there is some evidence that exposure to a certain environment alters the way people perceive and rate faces. For example, Webster et al. [4] tested Japanese students who had become residents in the US and found that the degree of the shift in ethnicity boundaries in face perception was correlated with the length of time these students lived in the US and also the degree of daily exposure to Caucasian faces. The authors conclude that natural stimulus variations are strong enough to evoke perceptual shifts in observers.

This emphasizes that adaptation is not only a construct investigated in psychophysical research but indeed has relevance with regard to what we perceive and experience in natural environments. In Western societies and the corresponding mass media women with a thin and preferably athletic body shape are most often idealized and slim women are illustrated to be more attractive, more desirable and more successful than women with larger body shapes [37]. There is also evidence that exposure to these idealized images induces and enhances body dissatisfaction in women [38], while the degree of internalization of this thin ideal even predicts body dissatisfaction [39], [40]. Strikingly, it was found that women who do not adopt this Western thin body ideal exhibit less body dissatisfaction and less often develop eating disorders [41], [42]. Glauert et al. [20] found that not only body normality but also body ideal could be easily altered in their participants due to exposure to thin and fat body shapes.

Given the facts that natural exposure to certain stimuli probably evokes perceptual biases [4] and that exposure to idealized or slim female bodies leads to a greater body dissatisfaction [38], a shift in perceived body ideal [20], and a perceptual bias for the own body shape as demonstrated in the present study, this might strongly contribute to the development and maintenance of body image disturbances in eating disorders. It would be very interesting to test whether women suffering from eating disorders (Anorexia nervosa and Bulimia nervosa) experience similar perceptual biases due to adaptation to thin and fat body shapes as found in the present study. The results could provide suggestions for the development of a systematic body exposure therapy, complementing existing forms of therapy.
